# Cryogenic conditioning of microencapsulated phase change material for thermal energy storage

**DOI:** 10.1038/s41598-020-75494-8

**Published:** 2020-10-27

**Authors:** G. V. N. Trivedi, R. Parameshwaran

**Affiliations:** Department of Mechanical Engineering, Birla Institute of Technology and Science-Pilani, Hyderabad Campus, Hyderabad, 500 078 India

**Keywords:** Mechanical engineering, Materials for energy and catalysis

## Abstract

Microencapsulation is a viable technique to protect and retain the properties of phase change materials (PCMs) that are used in thermal energy storage (TES) applications. In this study, an organic ester as a phase change material was microencapsulated using melamine–formaldehyde as the shell material. This microencapsulated PCM (MPCM) was examined with cyclic cryogenic treatment and combined cyclic cryogenic heat treatment processes. The surface morphology studies showed that the shell surfaces had no distortions or roughness after cryogenic treatment. The cryogenically conditioned microcapsules exhibited diffraction peak intensity shifts and crystal structure changes. The onset of melting for the nonconditioned and conditioned microcapsules were measured to be 8.56–9.56 °C, respectively. Furthermore, after undergoing the cryogenic and heat treatment processes, the PCM microcapsules had appreciable latent heat capacities of 39.8 kJ/kg and 60.7 kJ/kg, respectively. Additionally, the microcapsules were found to have good chemical stability after the cryogenic treatment. In addition, the cryogenically conditioned microcapsules were found to be thermally stable up to 128.9 °C, whereas the nonconditioned microcapsules were stable up to 101.9 °C. Based on the test results, it is obvious that the cryogenically conditioned microcapsules exhibited good thermal properties and are very desirable for cool thermal energy storage applications.

## Introduction

Energy storage technologies are gaining attention to address the global energy demand caused by the development of industrial and economic sectors. These technologies are able to fill the gap between energy demand and supply, thus improving energy utilization with minimal waste. They also possess the benefits of reducing greenhouse gases, reducing energy costs, providing energy security and conserving of fossil fuels. Among the various available technologies for energy storage, thermal energy storage is an eco-friendly technology that can be accomplished by sensible storage, latent storage and thermochemical energy storage^1,2^.

Phase change materials are latent TES materials that can store and release heat energy through liquid to solid or solid to liquid phase transitions at near isothermal conditions. These energy storage materials are widely used in building materials^3^, heat transfer fluids^4^, solar cooling^5^ and electronics cooling^6^.

There are three basic types of PCMs that are commercially available, namely, inorganic PCMs (e.g., salt hydrates), organic PCMs (e.g., paraffins, fatty acids and fatty acid esters) and eutectic PCMs (eutectic salts and solutions)^7^. Of the three types, organic PCMs are mostly preferred for cooling applications in buildings due to their excellent thermophysical properties^8^, congruent freezing and melting^9^, thermal stability^10^, low corrosiveness^11^, and thermal reliability^12,13^, in addition to other benefits^14^.

The selection and successful utilization of organic PCMs for cool thermal energy storage (CTES) applications have not yet been fully explored and are still limited by both macro- and microscale approaches. The issues of organic PCMs are related to their low thermal conductivity, supercooling degree, low heat transfer rate during freezing and melting, and leakage. Nevertheless, these limitations have been partially addressed by macro- and microencapsulation techniques^15–18^.

Various studies have reported the microencapsulation of PCMs with organic shell materials through the use of interfacial polymerization, emulsion polymerization, suspension polymerization, and in situ polymerization. Sari et al.^19^ microencapsulated various types of paraffin mixtures into a poly(methyl methacrylate) shell material via emulsion polymerization. These microcapsules were tested for 5000 thermal cycles and exhibited thermal reliability and good chemical stability between the core and shell materials. Su et al.^20^ microencapsulated the paraffin into melamine–formaldehyde using an in situ polymerization technique. They found that the utilization of the surfactant had a significant effect on the surface morphology and reported that the utilization of binary emulsifiers reduced the agglomeration of capsules.

Huang et al.^21^ microencapsulated n-dodecanol, a nonparaffin-based PCM, into a melamine–formaldehyde shell through in situ polymerization. Their findings showed that the polymer-based microcapsules showed good flexibility and rigidity under the applied load. Dhivya et al.^22^ encapsulated a eutectic-based PCM into a melamine–formaldehyde shell and reported that the microcapsules showed good thermal stability and retained their shape without any distortions, even after thermal cycling. Konuklu et al.^23^ microencapsulated capric acid into a melamine-urea–formaldehyde shell and demonstrated that the shell material showed good heat resistance and excellent sealant characteristics at a temperature of 95 °C.

In addition to amine-based shell materials, Sari et al.^24^ encapsulated various fatty acids, i.e., lauric acid, myristic acid, and capric acid, into polystyrene using emulsion polymerization and performed thermal reliability testing. The outcome of the study showed that the polystyrene shells exhibited good chemical stability with all fatty acids. Sami et al.^25^ also carried out a similar line of studies about lauric acid as the PCM and polystyrene as the shell and reported the effects of the shell-to-core ratio, stirring rate, and temperature on the thermal properties of the encapsulated PCM. Therefore, organic shell materials have the proven merits of excellent sealant characteristics, high durability, easy surface modification, and excellent chemical stability for long-term use.

However, they still lack in terms of the thermal stability and thermal conductivity compared to those of inorganic shells. In this context, a few research studies were performed recently by implementing nanoparticles onto the surface of organic shells to enhance the thermal properties of the microcapsules. Sarier et al.^26^ embedded silver nanoparticles from 28 to 58 nm in size into a urea–formaldehyde prepolymer solution during the preparation process and reported a substantial improvement in the thermal conductivity and thermal stability of n-alkane-based microcapsules. Zhuang et al. incorporated iron oxide, titanium dioxide, and zinc oxide nanoparticles into a urea–formaldehyde shell material and reported that the addition of the nanoparticles improved the thermal stability but resulted in irregular and roughened surfaces. In addition, ZnO^27^ and Fe_3_O_4_^28^ nanoparticles blended with organic shell materials also produced microcapsules with increased thermal stability.

From the viewpoint of the property enhancement in different kinds of materials, the temperature at which the materials are exposed has shown a considerable change in their inherent properties. This holds true for materials being subjected to either heat treatments or cold working conditions to attain tunable/desirable and adaptable properties. Similarly, cryogenic conditioning is a technique that involves the treatment of materials with liquid nitrogen (LN_2_) at a very cold temperature of − 196 °C (77.2 K).

Cryogenic treatment is widely applied to tool steels and carbides to improve their mechanical and thermal properties by means of promoting crystal transformations^29^. Likewise, the SnSb/C nanofibers treated under LN_2_ for 12 h showed an enhancement in their electrochemical performance along with crystal structure and morphological changes^30^. SiC nanoparticles treated for 24 h showed an increase in thermal conductivity and a decrease in specific heat^31^. Both studies reported that there was a significant reduction in the material sizes after the cryogenic treatment.

Additionally, it was observed that the agglomeration of TiC nanoparticles decreased significantly and the thermal conductivity increased after cryogenic treatment^32^. In addition to the studies conducted on the thermal properties, the mechanical response of ZnO nanowires under cryogenic treatment was also reported^33^. Very few studies have reported polymers and polymer composites treated at liquid nitrogen temperatures to increase and promote crystal changes and increase the strength of such materials^34,35^.

However, research studies on powder-based materials treated with cryogen (cryogenic liquid) are limited. Interestingly, one study demonstrated the thermomechanical behavior of microspheres comprising a PCM for cryogenic-temperature cold storage applications^36^. The results infer that the buckling of the shell and freezing time of the PCM microspheres were vital parameters in determining the heat transfer and charging efficacy of cold storage.

On the one hand, there is still tremendous potential for achieving tunable and adaptable thermal storage properties in organic PCMs at the microscale that are suitable for cooling applications. Hence, based on the literature and to the best of our knowledge, no studies have been reported thus far pertaining to the cryogenic treatment of microencapsulated PCMs (MPCMs) for CTES applications. Therefore, in this study, an attempt was made to explore the thermal storage capabilities of a microencapsulated organic ester PCM through a novel approach.

The present study is truly distinct from the past literature in that the as-synthesized PCM microspheres were actually treated using liquid nitrogen (LN_2_), followed by heat treatment, and once again, they were cryogenically conditioned. This was performed to achieve the desired thermal storage properties in the as-prepared cryogenically conditioned MPCMs (CMPCMs) for CTES applications.

The CMPCMs were further tested using appropriate characterization techniques. The surface morphology of the prepared CMPCM was observed using field emission scanning electron microscopy (FESEM) on an instrument equipped with an energy dispersive X-ray spectrometry (EDS). The effect of cryogenic treatment on the crystal structure and phase change characteristics were studied using X-ray diffraction (XRD) and differential scanning calorimetry (DSC) techniques, respectively. Furthermore, the chemical structure and thermal stability were characterized using Fourier transform infrared (FTIR) spectroscopy and thermogravimetric analysis (TGA), respectively, and the results are presented and discussed.

## Results

### Surface morphologies and elemental compositions

**Figure 1:**
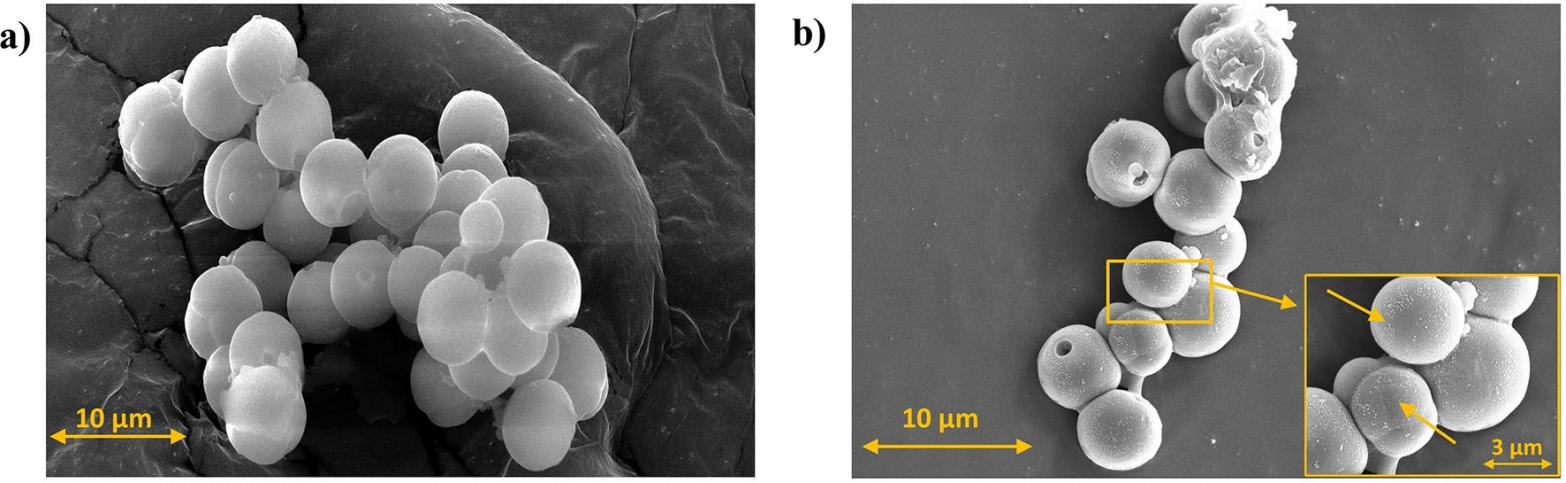
Surface morphology obtained from FESEM: (**a**) surface morphology of MPCM and (**b**) surface morphology of CMPCM1.

**Figure 2:**
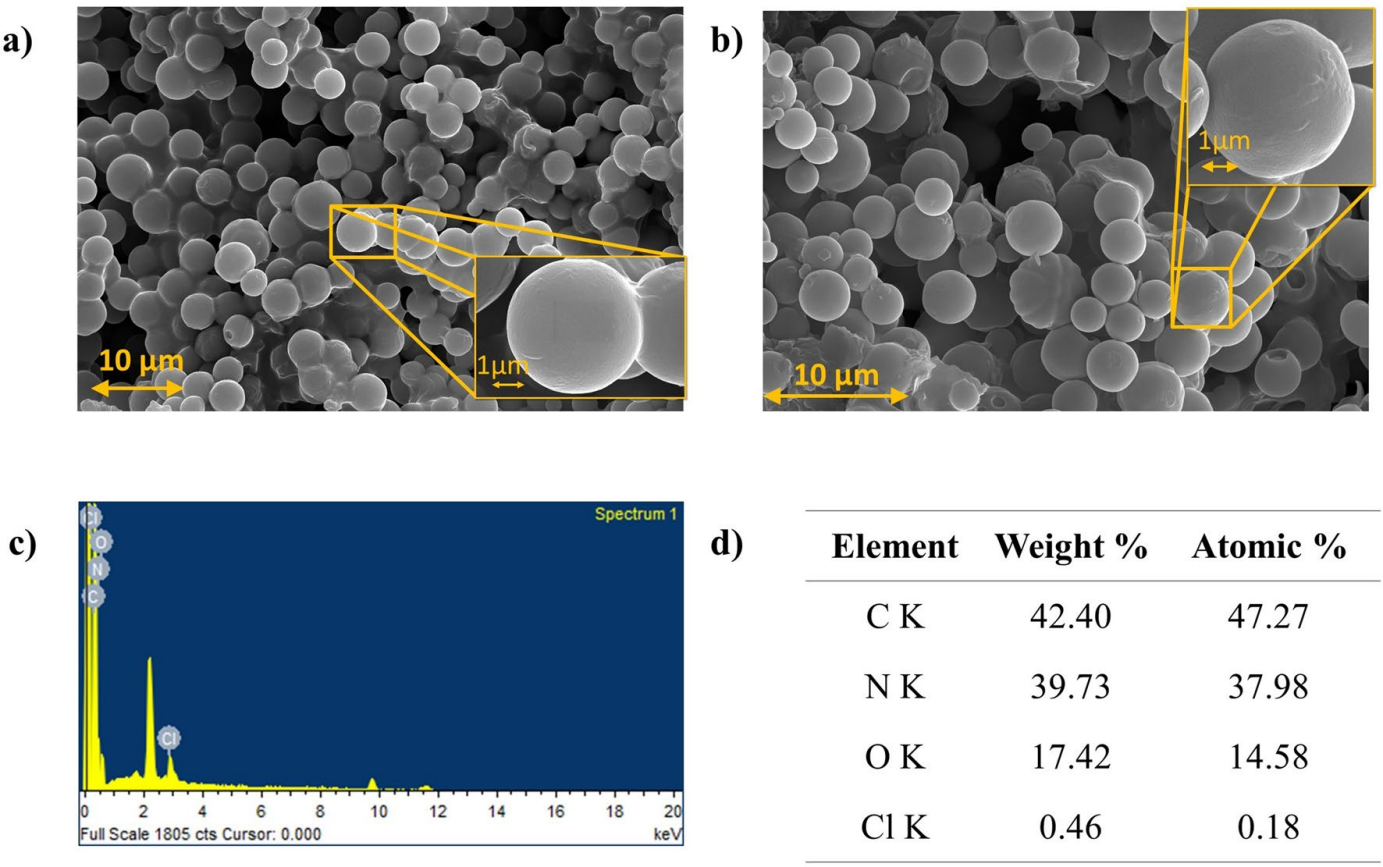
Surface morphology and elemental composition obtained from FESEM + EDS: (**a**) surface morphology of noncryogenically treated microcapsules, (**b**) surface morphology of CMPCM2, (**c**) EDS spectra of MPCM, and (**d**) concentrations as elemental atomic and weight percents.

The surface morphology of the as-prepared microcapsules are shown in Figs. 1a, 2a, and the cryogenically conditioned microcapsules are illustrated in Figs. 1b, 2b. The microcapsules prepared by in situ polymerization showed good sphericity with smooth shell surfaces. However, the microcapsules (in Fig. 1b) treated under LN_2_ for 4 h revealed the development of rough shell surfaces, as indicated in Fig. 1b, which was observed to be quite similar to the morphology of other reported cryogenically treated materials^30^.

The morphology of microcapsules treated under LN_2_ for 4 h, followed by heat treatment at 70 °C and further conditioning by LN_2_ for another 4 h is presented in Fig. 2b. The FESEM images infer that CMPCM2 had smooth shell surfaces without the development of rough shell surfaces. The obtained EDS spectra, element weights and atomic percentages are shown in Fig. 2c,d.

### Crystal structures

**Figure 3:**
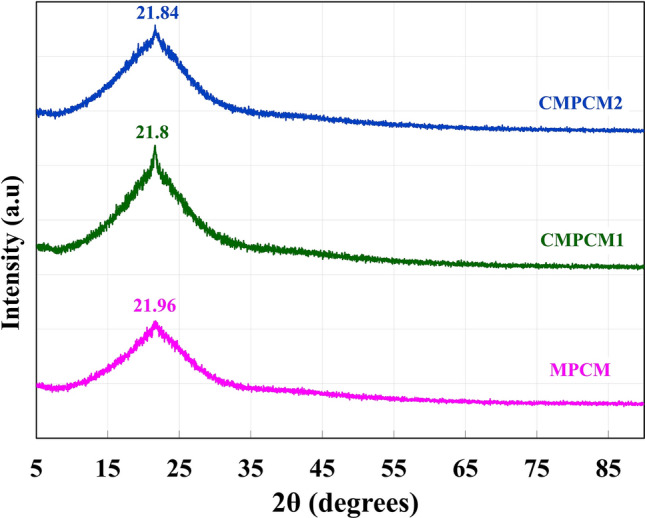
Crystal structures of MPCM, CMPCM1 and CMPCM2.

The crystal structures of MPCM, CMPCM1 and CMPCM2 obtained from XRD are presented in Fig. 3. The obtained results indicate that CMPCM1 and CMPCM2 underwent crystal structure changes and had marginal diffraction intensity shifts in comparison to those for MPCM. However, CMCPM2 prepared through the combined cryogenic and heat treatment processes exhibited a reduction in the sharp diffraction intensity compared to that of CMPCM1.

### Chemical structure

**Figure 4:**
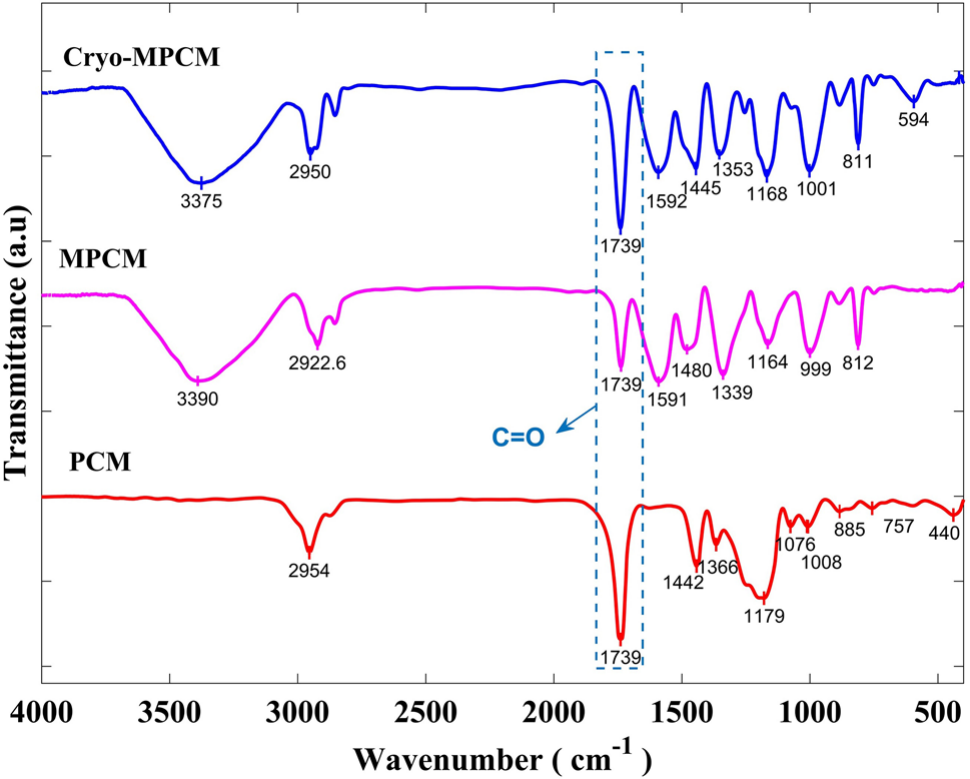
Chemical structure of PCM, MPCM, and Cryo-MPCM obtained from FTIR spectrometry.

Figure 4 illustrates the chemical structure of the ester-based PCM, MPCM and the cryogenically conditioned microcapsules (Cryo-MPCM). As the FTIR results for CMPCM1 and CMPCM2 overlapped with each other, only one curve is depicted to represent them and is labelled as Cryo-MPCM. The strong and sharp absorption at 1739 cm^−1^ was attributed to the C=O stretching vibration of the ester. The absorption peaks observed in the band from 1300 to 1000 cm^−1^ corresponded to the C-O stretching vibration of the ester. The absorption peaks in the range from 3300 to 3400 cm^−1^ corresponded to the stretching vibration of the N–H primary amine.

The broad and strong absorption peaks occurring near 1591 cm^−1^ were attributed to the bending of the N–H primary amine. The absorption spectra in the range from 1310 to 1360 cm^−1^ signified stretching of the aromatic tertiary amine (C-N). The absorption peak near 1164 cm^−1^ indicated the presence of C-N stretching of the aliphatic amine. The sharp absorption at 812 cm^−1^ indicated bending vibration of the triazine ring^37^.

### Phase change characteristics

**Figure 5:**
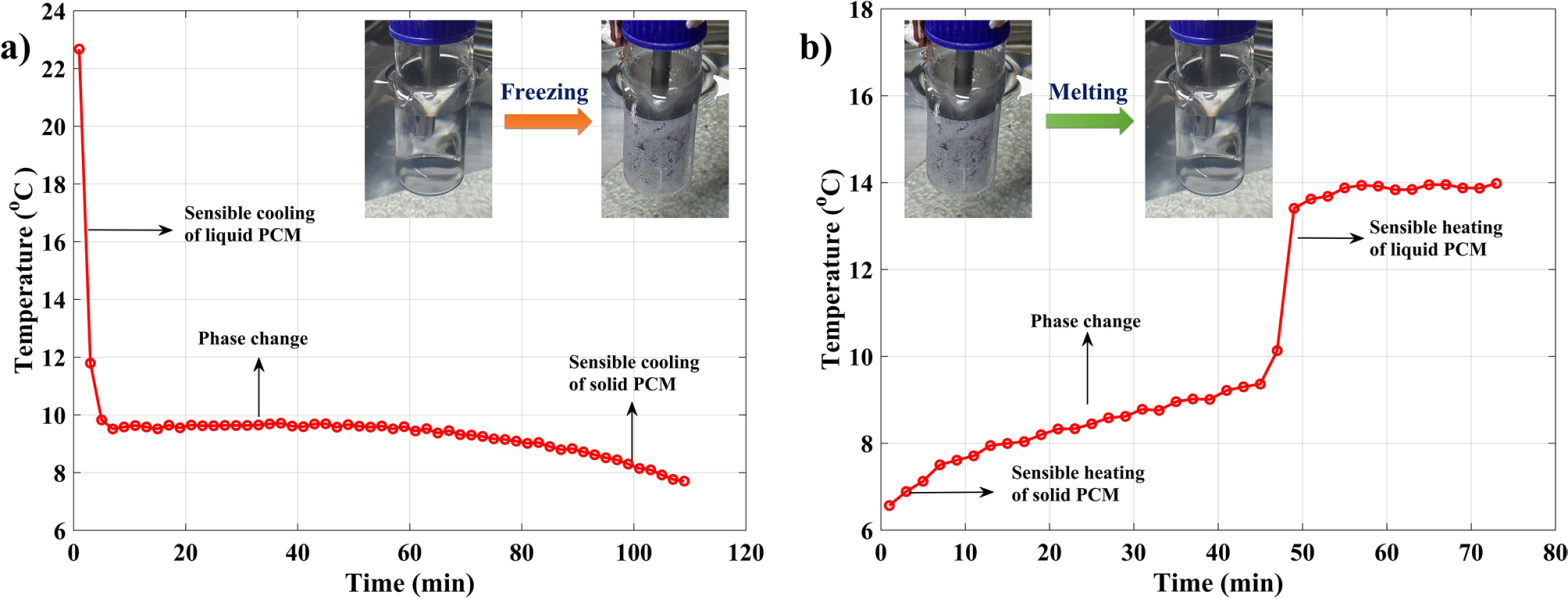
(**a**) Freezing curve of dimethyl adipate and (**b**) melting curve of dimethyl adipate.

The freezing and melting behavior of the obtained PCM was experimentally investigated using a thermostatic water bath, and the obtained temperature–time data are presented in Fig. 5a,b. The obtained results reveal that freezing (crystallization) of the ester-based PCM occurred in three stages. The pure PCM that existed in the liquid state at a temperature of 23 °C released sensible heat to the surrounding heat transfer fluid and the temperature was decreased gradually. Once the PCM reached its freezing temperature, the pure PCM started to crystallize, and the phase change from liquid to solid initiated at a temperature of approximately 9.5 °C.

The inset in Fig. 5a shows the crystallization that occurred at near isothermal conditions. The phase change process was followed by a sensible heat release from the crystalline PCM to achieve thermal equilibrium with the surrounding fluid. The obtained temperature profile is in good agreement with results reported in the literature^38^. In similar aspects, the melting of the crystalline PCM also underwent three stages, i.e., sensible heat absorption in the solid state, a solid-to-liquid phase transition, and sensible heat absorption in the liquid state, as shown in Fig. 5b. However, during the melting process, the phase transition occurred in the range from 7 to 9 °C.

**Figure 6:**
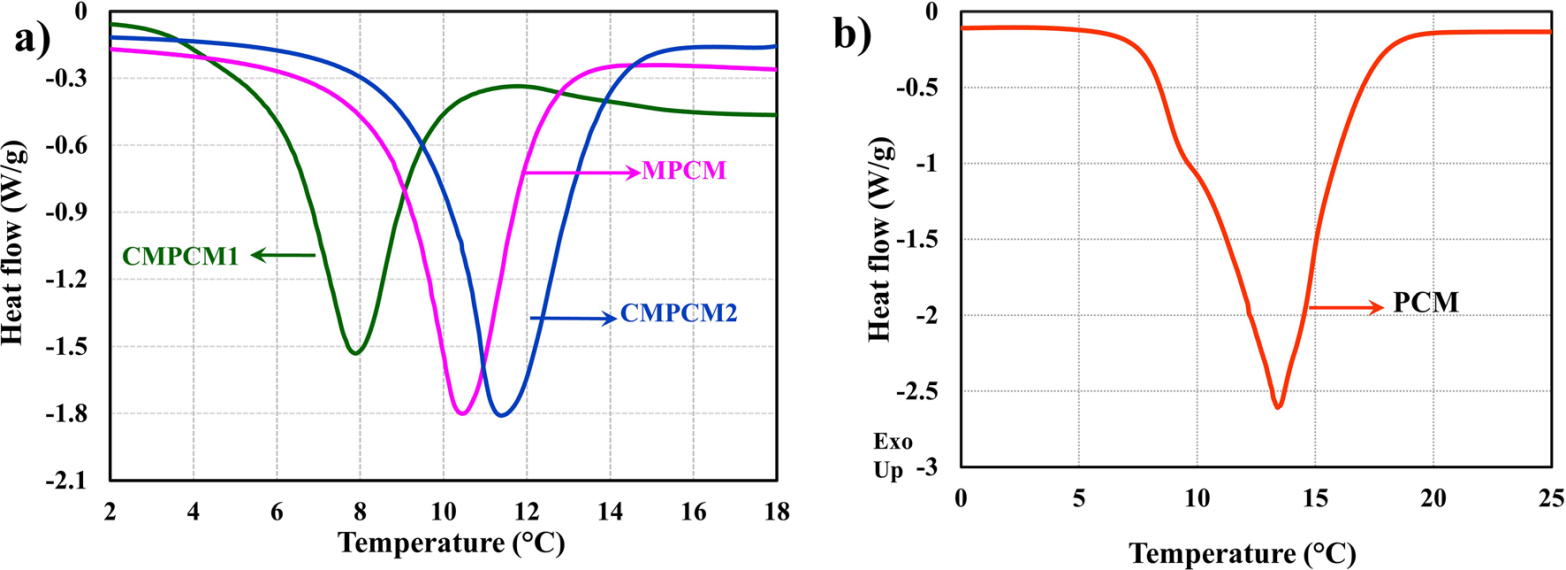
(**a**) DSC results of MPCM, CMPCM1 and CMPCM2 and (**b**) DSC results of PCM.

The phase change properties of MPCM, CMPCM1, CMPCM2 and pure PCM obtained using the DSC are presented in Fig. 6a,b and Table 1. The onset of melting of the ester-based pure PCM and MPCM were 9.24 °C and 8.56 °C, respectively, with a single endothermic heat flow thermograph, as shown in Fig. 6a, b. The latent heat of fusion for the pure PCM and MPCM were measured to be 153 kJ/kg and 48.3 kJ/kg, respectively.

**Table 1:** Thermal energy storage properties.

Sample	Phase change properties during melting		
	Onset temperature (°C)	Peak temperature (°C)	Latent heat (J/g)
PCM	9.24	13.4	153
MPCM	8.56	10.38	48.3
CMPCM1	5.9	7.94	39.8
CMPCM2	9.56	11.39	60.7

The as-synthesized and cryogenically conditioned microcapsules exhibited an endothermic peak that was found to be similar to that of the pure PCM. However, upon cryogenic treatment for 4 h, CMPCM1 exhibited a decrease in the onset of melting (5.9 °C) compared to that for the noncryogenically treated microcapsules (MPCM). On the other hand, the CMPCM2 obtained through cyclic LN_2_ conditioning and heating showed an onset melting point of 9.56 °C. Likewise, the latent heats of fusion for CMPCM1 and CMPCM2 were measured to be 39.8 kJ/kg and 60.7 kJ/kg, respectively.

### Thermal stability

**Figure 7:**
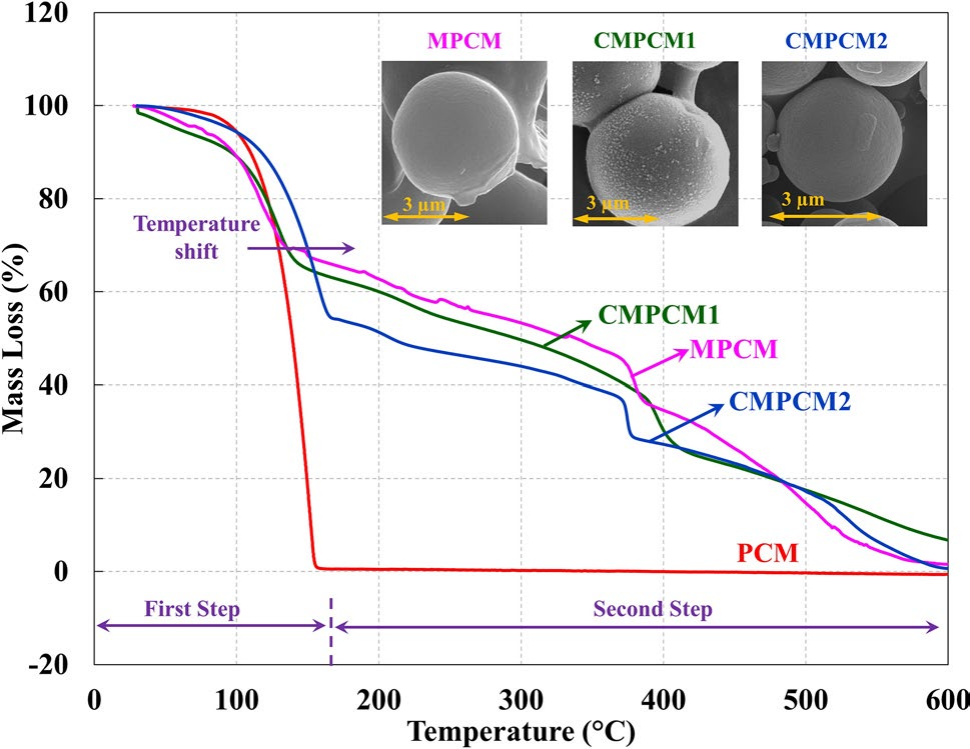
TGA results for PCM, MPCM, CMPCM1 and CMPCM2.

The thermal stability of PCM, MPCM, CMPCM1 and CMPCM2 was measured using TGA, and the results are shown in Fig. 7 and Table 2. The pure PCM displayed a single decomposition step without any traces of erosion on the testing crucible. The corresponding onset and end set temperatures were measured to be 130.8 °C and 156.8 °C, respectively. The MPCM, CMPCM1 and CMPCM2 curves showed a two-step decomposition with an increase in the decomposition temperature, which is in good agreement with reported results^7^.

**Table 2:** Thermal properties of PCM, MPCM, CMPCM1 and CMPCM2.

	First step				Second step			
	Onset temperature (°C)	Midpoint temperature (°C)	End set temperature (°C)	Mass loss (%)	Onset temperature (°C)	Midpoint temperature (°C)	End set temperature (°C)	Mass loss (%)
PCM	130.8	139.8	156.8	99.32	–		–	–
MPCM	101.9	112.6	130.3	31.03	372.9	375.9	383.9	15.82
CMPCM1	112.1	123.2	139.9	27.16	385.9	391.4	406.03	21.16
CMPCM2	128.6	141.5	163.6	45.7	370.7	371.9	373.4	16.98

The obtained onset decomposition temperatures are presented in Table 2 for cryogenically and noncryogenically treated samples. During the first degradation step in the range from 30 to 165 °C, water vapor absorbed by the shell surface evaporated, which was followed by a sudden mass loss due to elimination of PCM^21,39^.

The second step of mass decomposition in the temperature range from 165 to 600 °C for all the microcapsules corresponded to the melamine–formaldehyde shell. In this step, a continuous mass loss observed in the range from 165 to 350 °C was ascribed to the elimination of formaldehyde from the shell. Furthermore, an abrupt mass loss observed beyond 350 °C was due to the breakdown of the methylene bridges, followed by the degradation of the triazine ring^37^.

## Discussion

In this study, upon cryogenic conditioning the PCM present inside the shell underwent the freezing and melting processes, the density and volume changes during the phase change could have resulted in mechanical stress development at the core and interfaces of the polymer shell. These contraction forces were responsible for the fractures and microcracks and expected to be minimized by gradually conditioning MPCM from room temperature to the cryogenic temperature^40^. Upon doing so, CMPCM1 exhibited a surface morphology transformation from a smooth to rough surface^30^.

However, CMPCM2, which was obtained through cyclic cryogenic conditioning and heat treatment processes, retained smooth surfaces without any surface distortions, as shown in Fig. 2b. Thus, the combination of cryogenic and heat treatments prevented the shells of the CMPCM2 microcapsules from becoming rough and protected the shells from any premature cracking or surface distortions due to very low-temperature effects. In addition, it is evident from Fig. 1b that few microspheres in CMPCM1 contained an open shell structure, whereas for CMPCM2, no such open shell structures were observed.

The EDS results in Fig. 2d confirmed the presence of carbon (C), nitrogen (N), oxygen (O) and chlorine (Cl) elements, which were the constituents of the as-synthesized microcapsules. The presence of C and N as the major elements indicated the encapsulation of the ester-based organic PCM inside the amine shell material, which was formed through methylation and condensation processes^37^. The presence of C and O elements confirmed the existence of the PCM within the melamine–formaldehyde shell even after the cryogenic and heat treatment processes.

From the XRD results, it can be seen that the cryogenic treatment resulted in crystal structure changes and a slight increase in the crystallinity (for CMPCM1), which was similar to observations from studies related to cryogenic-treated polymers^36^. However, the peak intensity of CMPCM2 was quite low compared to that of CMPCM1, which could be attributed to its encapsulation properties. That is, CMPCM2 had low intensity diffraction peaks and a higher cracking resistance than CMPCM1^39^.

On the other hand, as the MPCM was subjected to the cryogenic temperature, the atomic level vibrations likely slowed , which could have increased the molecular bonding energy^41^. Due to this phenomenon, the core and shell elements of the microcapsules would have undergone thermal contractions. However, due to the different shrinkage rates of the core and the shell elements, internal stresses were expected to be induced.

Therefore, heat treatment of the CMPCM2 microcapsules after cryogenic conditioning facilitated relief of the internal stresses and resulted in grain refinement and particle alignment^30,40^. Moreover, the timely relief of the internal stresses resulted in intensity shifts and sharper diffraction peaks compared to those for the nonconditioned microcapsule. The chemical structure studies indicated that the as-prepared MPCM showed the characteristic absorption peak of the ester functional group at 1739 cm^−1^ along with primary amine absorption peaks at 3390 cm^−1^ and 1591 cm^−1^. Hence, the existence of the characteristic peaks of the ester-based PCM and the amine confirmed the formation of the melamine–formaldehyde shell over the PCM droplets, and the shell formed due to physical interactions without any chemical reactivity. Both CMPCM1 and CMPCM2 also exhibited similar characteristic peaks as those from MPCM, and thus, the chemical stability of the cryogenically conditioned and heat-treated microcapsules was sustained effectively.

The phase change characteristics of PCM, MPCM, CMPCM1 and CMPCM2, as shown in Fig. 6a,b, indicated that the change in the melting points could be attributed to motion of the PCM inside a confined space and an increase in the heat transfer surface area^42^. The difference in the latent heat capacities of microcapsules can be due to the lower shell strength, allowing some quantity of PCM to leak out of shell materials for MPCM and CMPCM1^43^. However, upon cyclic cryogenic conditioning and heat treatment, refinement of the shell material crystal structure could also have altered the shell material strength and avoided leakage of the PCM for CMPCM2.

A comparison of the thermal storage properties obtained in this study with respect to those in the literature is summarized in Table 3. Indeed, the phase change temperature and the encapsulation ratio of the microcapsules were in the appreciable range and therefore suitable for CTES applications.

**Table 3:** Comparison of the present study results with those in the literature.

Shell	PCM	Melting point (°C)	Encapsulation ratio (%)	References
Melamine–formaldehyde	1-Dodecanol	27.5	47–54	^21^
	Eutectic mixture	4–5	51–53	^22^
	Paraffin	47–48	29–65	^20^
Urea–formaldehyde	n-Hexadecane	15–18	37–52	^26^
Poly(melamine-urea–formaldehyde)	Decanoic acid	28–30	37–53	^23^
Polymethylmethacrylate	Stearic acid	55.3	52.2	^44^
	n-Octadecane	23–25	37–70	^45^
Polyurethane	Butyl stearate	21–22	70–74	^46^
Melamine–formaldehyde	Dimethyl adipate	5.9–9.5	26–39	Present study

Based on the thermal stability studies of the microcapsules, the first step of the decomposition process was ascribed to the breakdown of the saturated hydrocarbon ester groups into carbon monoxide, carbon dioxide and volatile low-molecular weight hydrocarbons^47^. This gasification process tended to increase the pressure within the microcapsules, which resulted in the rupture of the shells and thus, evaporation of the PCM from the microcapsules occurred.

Due to cryogenic conditioning, crystal structure changes enhanced the thermal stability of the shell, thereby increasing the ability of the shells to hold the internal pressures caused by the degradation of the core material (PCM). In addition, the heat treatment process carried out for the microcapsules after cryogenic conditioning also enhanced the thermal stability of the CMPCM2 shell^48^. That is, an increase in the onset of thermal decomposition of approximately 10% (112.1 °C) and 26% (128.6 °C) for CMPCM1 and CMPCM2, respectively, with respect to that for MPCM (101.9 °C) confirmed the viability of novel cryogenic and combined cryogenic heat treatment processes carried out on the microcapsules.

### In summary

In the present work, the thermal storage abilities of a new microencapsulated organic ester PCM were tested and characterized through a novel approach of cryogenic conditioning and combined cryogenic conditioning with heat treatment. Upon cryogenic conditioning, the microcapsules exhibited a good surface morphology without developing any microcracks. The XRD results of the CMPCMs showed a change in the crystal structure with intensity shifts and a marginal increase in crystallinity. The CMPCMs exhibited a slight change in the onset melting points; however, their latent heat storage capacities were found to be appropriate for the CTES application. MPCM, CMPCM1 and CMPCM2 were chemically stable according to the FTIR studies. Furthermore, compared with that for MPCM, the thermal stabilities of CMPCM1 and CMPCM2 were found to be enhanced by 10% and 26% upon cryogenic and combined cryogenic heat treatment processes, respectively.

## Methods

### Materials

To synthesize the microcapsules, dimethyl adipate (DMA), an organic ester, was procured from Alfa Aesar and utilized as the PCM. The melamine–formaldehyde shell precursors, pure-grade melamine (C_3_H_6_N_6_) and formaldehyde (HCHO) 37–38% w/w were purchased from Loba Chemie and Finar Ltd., respectively. The nonionic surfactants sorbitan monostearate (span) 60 and polysorbate (tween) 20 were purchased from SRL Pvt. Ltd. Ammonium chloride (NH_4_Cl), which served as a nucleating agent, was obtained from SD Fine-Chem Limited. Sodium hydroxide (NaOH) was utilized as a pH buffer during methylation and was procured from SD Fine-Chem Limited. Deionized water (DW) obtained from Milli-Q was used as the solvent. All the procured chemicals were utilized without any further purification.

### Preparation of MPCM

Microcapsules with melamine–formaldehyde as the shell and DMA as the core were prepared using approaches in previously reported studies^49^. Typically, DMA, Tween 20, span 60, and DW were mixed in a low-foam beaker and homogenized to form a stable oil-in-water (O/W) emulsion. Simultaneously, in another beaker, a prepolymer solution was prepared using melamine, formaldehyde solution and DW. The solution pH was adjusted to 11–12 using aqueous NaOH of 0.05 M, and stirring was continued until the solution became transparent.

**Figure 8:**
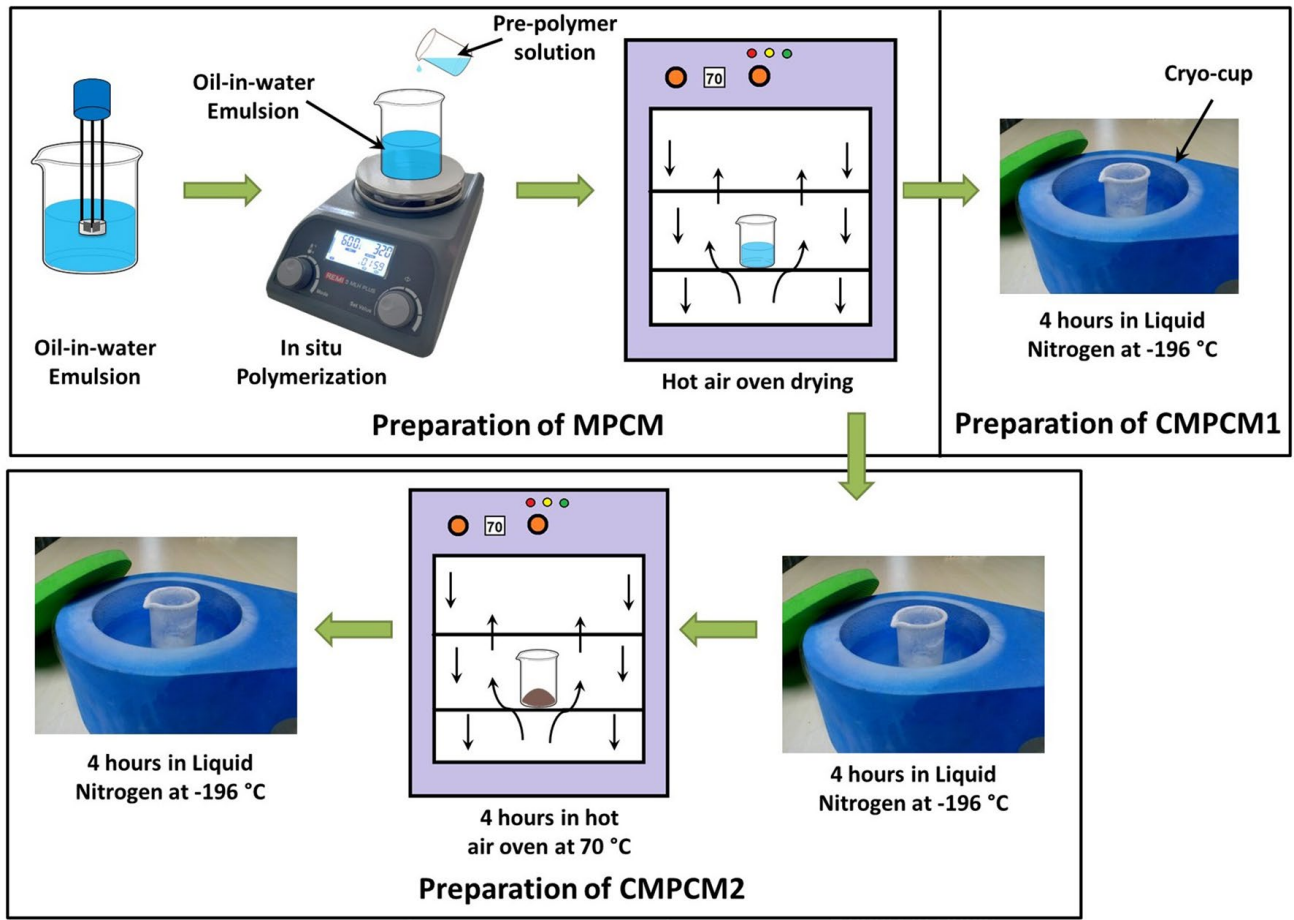
Schematic representation of the preparation of MPCM, CMPCM1 and CMPCM2.

This transparent solution was added dropwise into the O/W emulsion by maintaining continuous stirring with a magnetic stirrer (Make: REMI Laboratory Instruments). Ammonium chloride diluted in DW was added, and stirring was continued for 45 min. To remove the solvent and to obtain microcapsules in powder form, they were dried at a temperature of 70 °C using a hot air oven for 16 h to obtain MPCM, as illustrated in Fig. 8.

### Cryogenic treatment of MPCM

In this unique procedure, the cryogenic conditioning of the microcapsules was carried out in two way: (i) a portion of the microcapsules obtained from the hot air oven was treated in a cryo-cup (Make: SPEARLAB Cryogenic Products) containing LN_2_ at -196 °C for 4 h followed by curing to room temperature (30 °C) and (ii) the combined cryogenic and heat treatments of the remaining portion of the microcapsules were obtained from the hot air oven. The cryogenically conditioned microcapsules were designated as CMPCM1 and CMPCM2 before and after treatment procedures mentioned above, respectively.

For the case of the CMPCM2 preparation illustrated in Fig. 8, the MPCM capsules were first deep cooled in a cryo-cup containing LN_2_ at -196 °C for 4 h. Then, the conditioned microcapsules were allowed to warm to room temperature naturally, and they were placed in a hot air oven at 70 °C for 4 h. Then, the heat treated microcapsules were allowed to cool to room temperature and again placed in the cryo-cup containing LN_2_ for another 4 h. Finally, the CMPCM2 capsules were naturally cured to room temperature (30 °C).

### Instrumentation and characterization

The surface morphology and elemental composition of the microcapsules were investigated using FESEM (APERO-S, FEI) on an instrument equipped with energy dispersive X-ray spectrometry (EDS) operating in high-vacuum mode with an accelerating voltage of 20 kV. The microcapsules were coated with a gold–palladium alloy for analysis purposes.

The crystal structures of the microcapsules were recorded using X-ray diffraction (ULTIMA IV, RIGAKU) with Cu Kα (λ = 1.54060 A^o^) radiation, a 40 kV voltage, 30 mA current and scanning rate of 2°/min. The chemical structure was studied using FTIR spectrometry (FT/IR-4200, JASCO) on an instrument that was capable of recording the frequency over a range from 4000 to 400 cm^−1^.

The freezing and melting behaviors of the obtained PCM were experimentally investigated using a thermostatic water bath containing cooling and heating units equipped with an electric stirrer. The water bath temperatures were maintained at 6 °C during the freezing process and 14 °C during the melting process. A J-type thermocouple was centered in the PCM container to record the temperature changes during the freezing and melting processes using an Agilent 34972A data logger. The phase change properties of the microcapsules were measured using DSC (SHIMADZU) and subjected to heating and cooling cycles from -30 to + 30 °C at a rate of 5 °C/min under a nitrogen atmosphere.

Thermal stability studies were performed using a TG-60 (SHIMADZU) instrument. The microcapsule samples were heated from 30 to 600 °C at 10 °C/min under a nitrogen atmosphere.

## Conclusion

In this study, cryogenic conditioning of a microencapsulated organic ester PCM was performed to understand the very low-temperature effects on the thermal properties of the PCM microspheres. The FESEM studies showed that after cryogenic treatment, the surfaces of the microspheres were smooth without any surface distortions or microcracks. The crystal structure results revealed that the peak intensities of CMPCM1 and CMPCM2 exhibited minor shifts, and there was a difference in their crystal structures, which was attributed to the timely release of the internal stresses.

Moreover, the DSC results indicated latent heat potentials of 39.8 kJ/kg and 60.7 kJ/kg for CMPCM1 and CMPCM2, respectively, which indicates that they are feasible for the CTES application. In addition, the increase in the onset thermal decomposition temperature for CMPCM1 and CMPCM2 compared to that for MPCM (101.9 °C) was found to be 10% (112.1 °C) and 26% (128.6 °C), respectively, by cryogenic conditioning of the microcapsules. In total, the experimental results suggest that the as-synthesized CMPCM with good thermal properties can be considered a viable candidate for the CTES application.
